# Trace amounts of antibiotic altered metabolomic and microbial profiles of weaned pigs infected with a pathogenic *E. coli*

**DOI:** 10.1186/s40104-022-00703-5

**Published:** 2022-05-09

**Authors:** Kwangwook Kim, Cynthia Jinno, Peng Ji, Yanhong Liu

**Affiliations:** 1 Department of Animal Science, University of California, Davis, CA 95616 USA; 2 Department of Nutrition, University of California, Davis, CA 95616 USA

**Keywords:** Carbadox, Colon microbiota, Enterotoxigenic *Escherichia coli*, Metabolomics, Weaned pigs

## Abstract

**Background:**

Our previous study has shown that supplementation of trace amounts of antibiotic exacerbated the detrimental effects of enterotoxigenic *E. coli* (ETEC) infection and delayed the recovery of pigs that may be associated with modified metabolites and metabolic pathways. Therefore, the objective of this study was to explore the impacts of trace levels of antibiotic (carbadox) on host metabolic profiles and colon microbiota of weaned pigs experimentally infected with ETEC F18.

**Results:**

The multivariate analysis highlighted a distinct metabolomic profile of serum and colon digesta between trace amounts of antibiotic (TRA; 0.5 mg/kg carbadox) and label-recommended dose antibiotic (REC; 50 mg/kg carbadox) on d 5 post-inoculation (PI). The relative abundance of metabolomic markers of amino acids, carbohydrates, and purine metabolism were significantly differentiated between the TRA and REC groups (q < 0.2). In addition, pigs in REC group had the highest (*P* < 0.05) relative abundance of *Lactobacillaceae* and tended to have increased (*P* < 0.10) relative abundance of *Lachnospiraceae* in the colon digesta on d 5 PI. On d 11 PI, pigs in REC had greater (*P* < 0.05) relative abundance of *Clostridiaceae* compared with other groups, whereas had reduced (*P* < 0.05) relative abundance of *Prevotellaceae* than pigs in control group.

**Conclusions:**

Trace amounts of antibiotic resulted in differential metabolites and metabolic pathways that may be associated with its slow responses against ETEC F18 infection. The altered gut microbiota profiles by label-recommended dose antibiotic may contribute to the promotion of disease resistance in weaned pigs.

**Supplementary Information:**

The online version contains supplementary material available at 10.1186/s40104-022-00703-5.

## Background

Trace amounts of antibiotics are emerging contaminants of environmental concern due to their potential risks on non-target organisms and the spread of bacterial resistance [1]. The excessive and imprudent use of human and veterinary antibiotics significantly contributes to a continuous release of antibiotics into the environment, thus, a variety of trace concentrations of antibiotics have been detected in surface water, wastewater, soil, air, and dust [2–5]. Exposure to trace levels of antibiotics may cause unexpected adverse effects on the host, such as allergic reactions, disruption of digestive system function, and shaping the metabolites and microbial community [6, 7]. In particular, exposure to trace amounts of antibiotics at early life can result in the alteration of microbiota and metabolic networks, which further accelerate the possible development of resistant strains [8, 9]. Previous studies in mice have shown that subtherapeutic concentrations of penicillin exposure at early life interfered with the development of the intestinal immune system [10] and induced metabolic changes due to altered intestinal microbiota [11].

Our recent study has shown that trace amounts of antibiotic in feed exacerbated the detrimental effects of enterotoxigenic *E. coli* (ETEC) infection by increasing diarrhea and systemic inflammation in weanling pigs [12]. The exact mechanisms for the exacerbation of ETEC infection by trace amounts of antibiotic has not been determined, but it has been suggested that trace concentrations of antibiotics can act as signaling molecules to trigger specific bacterial responses [13]. Therefore, the objective of this study was to explore the impacts of feeding trace levels of antibiotic on host metabolic profiles and colon microbiota of weaned pigs experimentally infected with ETEC F18.

## Materials and methods

### Animals, housing, experimental design, and diet

The protocol for this study was reviewed and approved by the Institutional Animal Care and Use Committee at the University of California, Davis (IACUC #19322). Samples analyzed here were obtained as described in a previously published study by Kim et al. [12]. Briefly, 34 weanling pigs (crossbred; initial BW: 6.88 ± 1.03 kg) with an equal number of gilts and barrows were randomly assigned to one of three treatments in a randomized complete block design with body weight within sex and litter as the blocks and pig as the experimental unit. The 3 dietary treatments included: 1) the complex nursery basal diet (control; CON), 2) addition of 0.5 mg/kg carbadox (trace amounts of antibiotic; TRA) to the basal diet, or 3) addition of 50 mg/kg carbadox (label-recommended dose of antibiotic; REC) to the basal diet. All diets were formulated to meet pig nutritional requirements and provided as mash form throughout the experiment.

After 7 d of adaptation, all pigs were orally inoculated with 3 mL of ETEC F18 using a disposable Luer-lock syringe for 3 consecutive days from d 0 post-inoculation (PI). The ETEC F18 was originally isolated from a field disease outbreak by the University of Illinois Veterinary Diagnostic Lab (isolate number: U.IL-VDL # 05–27,242) and expresses heat-labile toxin (LT), heat-stable toxin b (STb), and Shiga-like toxin (SLT-2). The ETEC F18 inoculums were provided at 10^10^ colony-forming unit (CFU) per 3 mL dose in phosphate-buffered saline (PBS). This dose caused mild diarrhea in the current study as reported in Kim et al. [12], which is consistent with our previous published researches [14–16]. Growth performance, blood profiles, and immune responses were also reported in previous work [12].

### Sample collections

Sixteen pigs (6 pigs in CON, 4 pigs in TRA, and 6 pigs in REC) were randomly selected and euthanized on d 5 PI near the peak of infection, and the remaining pigs (6 pigs in CON, 5 pigs in TRA, and 7 pigs in REC) were euthanized at the end of the experiment (d 11 PI) that was the recovery period of the infection. The selection of necropsy time was based on the results of clinical observations and immune response parameters that were reported in previously published research using the same ETEC strain and inoculation dose [15, 16]. Before euthanasia, pigs were anesthetized with a 1-mL mixture of 100 mg telazol, 50 mg ketamine, and 50 mg xylazine (2:1:1) by intramuscular injection. After anesthesia, intracardiac injection with 78 mg sodium pentobarbital (Vortech Pharmaceuticals, Ltd., Dearborn, MI, USA) per 1 kg of BW was used to euthanize each pig. Blood samples were collected from the jugular vein of all pigs without EDTA to yield serum before ETEC challenge (d 0), and on d 5, and 11 PI. Serum samples were collected by centrifuging approximately 5 mL of whole blood samples at 20 °C at 1500 × *g* for 15 min and immediately stored at − 80 °C until untargeted metabolomics analysis. Colon digesta were collected from the distal colon of pigs on d 5 and 11 PI and immediately snap-frozen in liquid nitrogen and stored at − 80 °C for untargeted metabolomics and microbiome analysis.

### Untargeted metabolomics analysis

The untargeted metabolomics analysis was performed by the NIH West Coast Metabolomics Center using gas chromatography (Agilent 6890 gas chromatograph controlled using Leco ChromaTOF software version 2.32, Agilent, Santa Clara, CA, USA) coupled with time-of-flight mass spectrometry (GC/TOF-MS) (Leco Pegasus IV time-of-flight mass spectrometer controlled using Leco ChromaTOF software version3 2.32, Leco, Joseph, MI, USA). Metabolite extraction was performed following procedures described previously [17]. Briefly, frozen colon digesta samples (approximately 10 mg) were homogenized using a Retsch ball mill (Retsch, Newtown, PA, USA) for 30 s at 25 times/s. After homogenization, a prechilled (− 20 °C) extraction solution (isopropanol/acetonitrile/water at the volume ratio 3:3:2, degassed with liquid nitrogen) was added at a volume of 1 mL/20 mg of sample. Samples were then vortexed and shaken for metabolite extraction. After centrifugation at 12,800 × *g* for 2 min, the supernatant was collected and divided into two equal aliquots and concentrated at room temperature for 4 h in a cold-trap vacuum concentrator (Labconco Centrivap, Kansas City, MO, USA). To separate complex lipids and waxes, the residue was re-suspended in 500 μL of 50% aqueous acetonitrile and centrifuged at 12,800 × *g* for 2 min. The resultant supernatant was collected and concentrated in the vacuum concentrator. Dried sample extracts were derivatized and mixed with internal retention index markers (fatty acid methyl esters with the chain length of C8 to C30). The samples were injected for GC/TOF analysis, and all samples were analyzed in a single batch. Data acquisition by mass spectrometry and mass calibration using FC43 (perfluorotributylamine) before starting analysis sequences. Metabolite identifications was performed based on the two parameters: 1) Retention index window ± 2000 U (around ± 2 sec retention time deviation), and 2) Mass spectral similarity plus additional confidence criteria as detailed below (Data analysis). A detailed methodology for data acquisition and metabolite identification described in a previously published article by Fiehn et al. [17].

### Gut microbiota in distal Colon

Bacterial DNA was extracted from digesta samples using the Quick-DNA Fecal/Soil Microbe Kit (Zymo Research, Irvine, CA, USA) following the manufacturer’s instructions. Extracted bacterial DNA was amplified with PCR, targeting the V4 region of the 16S rRNA gene with primers 515 F (5′- XXXXXXXX*GT*GTGCCAGCMGCCGCGGTAA-3′) with an 8 bp barcode (X) and Illumina adapter (*GT*) and 806 R (5′-GGACTACHVGGGTWTCTAAT-3′) [18]. Amplification included thermocycling conditions of 94 °C for 3 min for denaturation, 35 cycles of 94 °C for 45 s, 50 °C for 1 min, 72 °C for 1.5 min, and 72 °C for 10 min (final elongation). To reduce PCR bias, each sample was amplified in triplicate. Each PCR reaction included 2 μL of template DNA, 0.5 μL (10 μmol/L) of barcoded forward primer, 0.5 μL (10 μmol/L) of reverse primer, 12.5 μL of GoTaq 2X Green Master Mix (Promega, Madison, WI, USA), and 9.5 μL of nuclease-free water. The triplicate PCR products were pooled and subjectively quantified based on the brightness of the bands on a 2% agarose gel with SYBR safe (Invitrogen Co., Carlsbad, CA, USA). All amplicons were then pooled at equal amounts and further purified using the QIAquick PCR Purification Kit (Qiagen, Hilden, Germany). The purified library was submitted to the UC Davis Genome Center DNA Technologies Core for 250 bp paired-end sequencing on the Illumina MiSeq platform (Illumina, Inc. San Diego, CA, USA).

The software sabre (https://github.com/najoshi/sabre) was used to demultiplex and remove barcodes from raw sequences. Sequences were then imported into Quantitative Insights Into Microbial Ecology 2 (QIIME2; version 2018.6) for downstream filtering and bioinformatics analysis [19, 20]. Plugin q2-dada2 [21] was used for quality control and constructing features. Taxonomic classification was assigned using the feature-classifier plugin trained with the SILVA rRNA database 99% operational taxonomic units (OTU), version 132 [22, 23].

### Data analysis

The metabolomics data were analyzed using different modules of a web-based platform, MetaboAnalyst 5.0 (https://www.metaboanalyst.ca) [24]. Data were filtered for peaks with detection rates less than 30% of missing abundances and normalized using logarithmic transformation and auto-scaling. Mass univariate analysis was performed using one-way ANOVA followed by Tukey’s post hoc test (adjusted *P* ≤ 0.05). Fold change analysis and *t*-tests were also conducted to determine the fold change and significance of each identified metabolite. Statistical significance was declared at a false discovery rate (FDR, Benjamini and Hochberg correction; q) q < 0.2 and fold change > 2.0. Partial least squares discriminant analysis (PLS-DA) was carried out to further identify discriminative variables (metabolites) among the treatment groups. Pathway analysis and metabolite set enrichment analysis were performed on identified metabolites that had a Variable Importance in Projection (VIP) score > 1.

Data visualization and statistical analysis for colon microbiota were conducted using R (version 3.6.1). Two alpha diversity indices, Chao1 and Shannon, were calculated using the phyloseq package. Relative abundance was calculated using the phyloseq package and visualized using the ggplot2 package in R. Relative abundance data were aggregated at various taxonomical levels. Shapiro-Wilk normality test and Bartlett test were used to verify normality and constant variance, respectively, in alpha diversity and relative abundance. Shannon index was analyzed using ANOVA with the statistical model, including sample collection days within treatment as fixed effects. Significance in Chao1 index and relative abundance was observed using Kruskal-Wallis rank-sum test followed by a Conover test for multiple pairwise comparisons using the agricolae package. Beta diversity was calculated based on the Bray-Curtis dissimilarity for principal coordinates analysis (PCoA). The homogeneity of multivariate dispersions was tested by the vegan package using the betadisper function, before the adonis function was used to calculate PERMANOVA with 999 replicate permutations. Statistical significance and tendency were considered at *P* < 0.05 and 0.05 ≤ *P* < 0.10, respectively.

## Results

### Metabolite profiles in serum

**Fig. 1:**
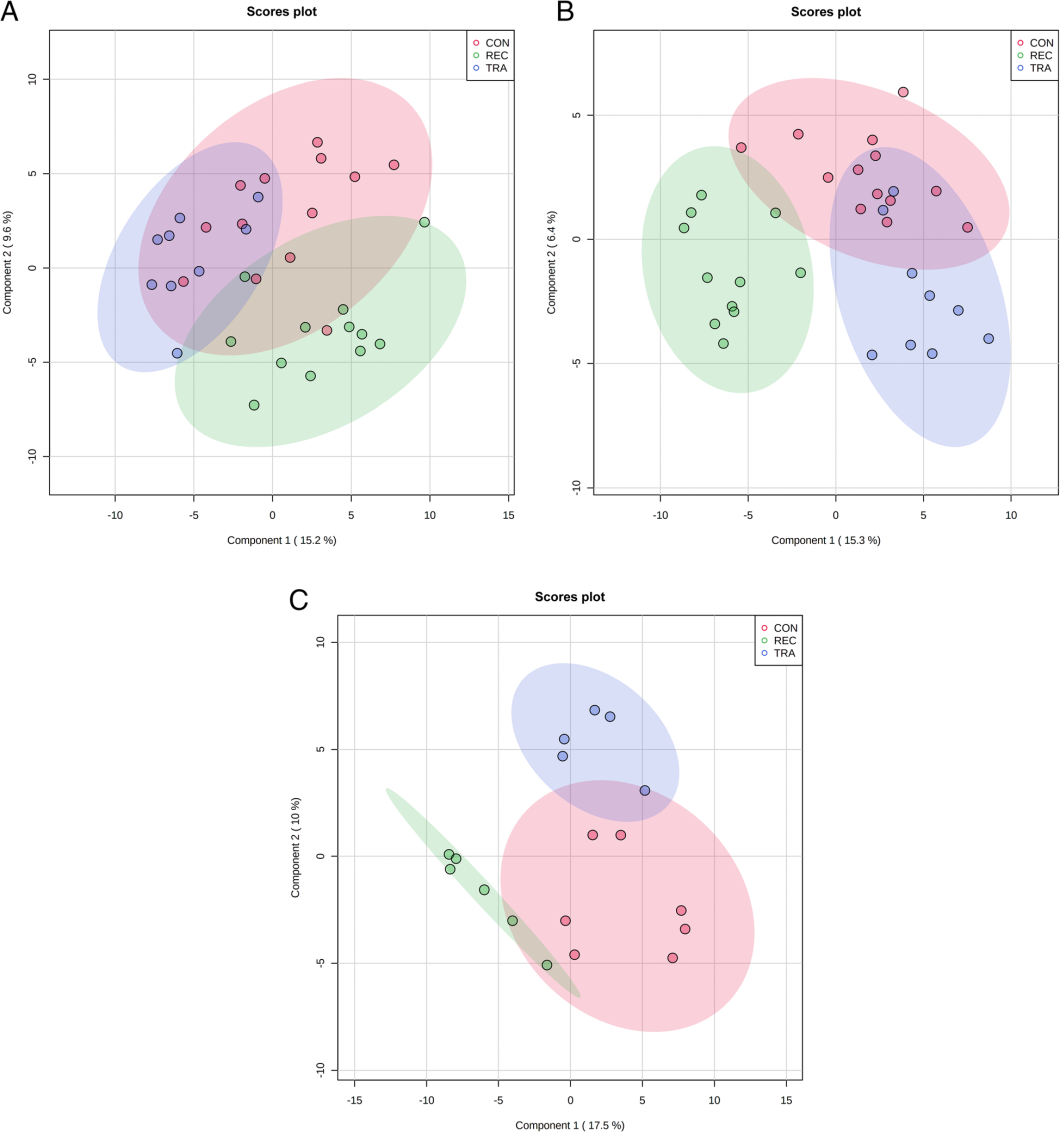
Partial Least Squares Discriminant Analysis (PLS-DA) 2D score plot of the metabolites in serum showed separated clusters between the TRA and REC groups on d 0 before ETEC challenge (**A**), d 5 (**B**) and 11 (**C**) PI. ● = CON (Control); ● = TRA (Trace amounts of antibiotic); ● = REC (Label-recommended dose of antibiotic). Shaded areas in different colors represent in 95% confidence interval

A total of 354 (134 identified and 220 unidentified) metabolites were detected in serum samples. Based on VIP scores and relative abundance, 3 metabolites (fructose, mannonic acid, and propyleneglycol) were up-regulated by TRA, compared with the pigs in REC on d 0 before ETEC challenge (Table 1). Supplementation of REC changed the abundances of 6 metabolites (2 up-regulated and 4 down-regulated) compared with CON, while REC changed 16 metabolites (6 up-regulated and 10 down-regulated) in comparison with TRA on d 5 PI. On d 11 PI, chenodeoxycholic acid was enriched, while glycerol and inositol-4-monophosphate were reduced in the CON group compared with REC. Pigs in TRA had greater chenodeoxycholic acid than pigs in REC, but 5 metabolites (p-tolyl glucuronide, glycerol, mannitol, 2-ketoisocaproic acid, and inositol-4-monophosphate) were decreased in pigs supplemented with TRA compared with pigs in REC. No differential metabolites were identified when comparing CON vs. TRA throughout the experiment. Based on the identified metabolites, a PLS-DA score plot with 95% confidence ranges (shaded areas) showed a clear separation between the TRA and REC groups throughout the experiment (Fig. 1). To further explore the metabolic profile differences among two dietary treatments, PLS-DA was performed for the following comparisons: (1) TRA vs. REC, and (2) CON vs. REC on d 0 before ETEC challenge, d 5 and d 11 PI. The score plot again distinguished the TRA from the REC, and also revealed the metabolic profile differences between CON and REC (Fig. S1).

**Table 1:** Serum metabolites that differed among the dietary treatment groups^1^

Metabolite	Fold change^1^	VIP^2^	FDR^3^
TRA^4^ vs. REC^5^, d 0 before ETEC challenge			
Fructose	2.13	1.88	0.108
Mannonic acid	2.21	2.01	0.083
Propyleneglycol	2.38	1.76	0.122
CON^﻿6^ vs. REC, d 5 post-inoculation			
Mannitol	0.23	1.48	0.115
Inosine	0.41	1.63	0.076
Glycerol	2.09	1.79	0.045
Galactonic acid	2.26	1.65	0.076
Propyleneglycol	2.51	1.47	0.119
Shikimic acid	2.64	1.86	0.036
TRA vs. REC, d 5 post-inoculation			
2-hydroxyvaleric acid	0.24	2.13	0.001
P-hydroxylphenyllactic acid	0.30	1.10	0.145
Pipecolinic acid	0.38	1.58	0.024
1-methylhydantoin	0.40	1.51	0.031
Histidine	0.45	1.98	0.002
Creatine	0.46	1.51	0.031
Myo-inositol	2.01	1.93	0.002
Guanine	2.03	1.83	0.005
Oleic acid	2.03	1.18	0.114
Montanic acid	2.05	1.57	0.024
Galactonic acid	2.11	1.42	0.046
Hypoxanthine	2.14	1.80	0.006
Glycerol	3.26	1.35	0.067
Propyleneglycol	4.04	2.00	0.002
Shikimic acid	4.47	1.63	0.020
Taurine	5.58	1.27	0.082
CON vs. REC, d 11 post-inoculation			
Glycerol	0.33	1.74	0.181
Inositol-4-monophosphate	0.48	1.92	0.165
Chenodeoxycholic acid	3.01	1.84	0.171
TRA vs. REC, d 11 post-inoculation			
P-tolyl glucuronide	0.26	2.10	0.106
Dlycerol	0.30	1.84	0.195
Mannitol	0.30	1.90	0.158
2-ketoisocaproic acid	0.45	1.90	0.158
Inositol-4-monophosphate	0.48	2.08	0.106
Chenodeoxycholic acid	4.67	2.04	0.109

^1^Fold change values less than one indicate that the differential metabolites were reduced in the CON compared to REC or TRA compared to REC, respectively

^2^*VIP* Variable Importance in the projection

^3^*FDR* False discovery rate

^4^*TRA* Trace amounts of antibiotic

^5^*REC* Label-recommended dose of antibiotic

^6^*CON* Control

**Fig. 2:**
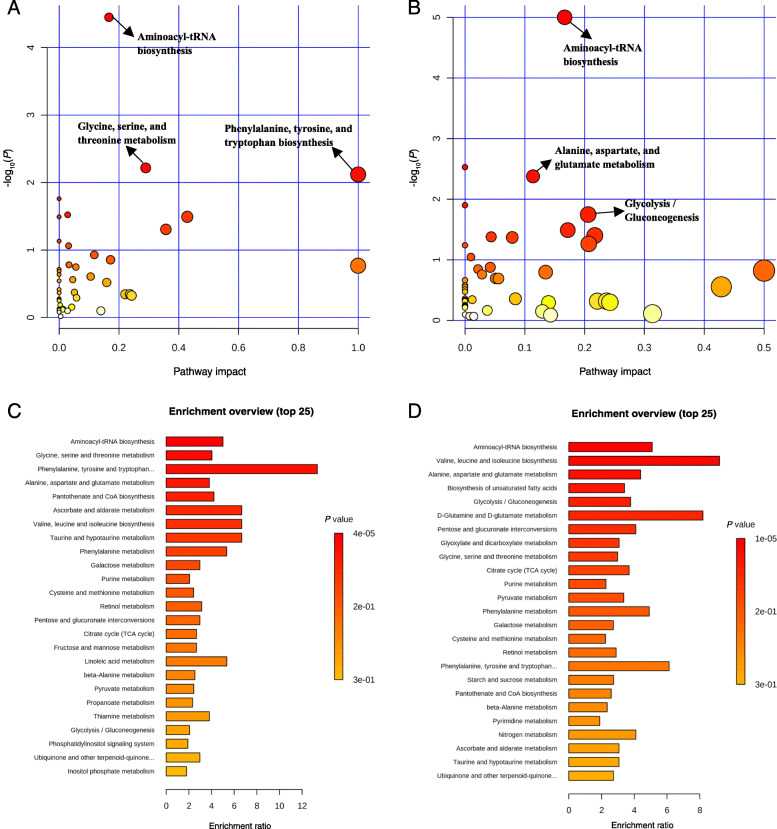
Significantly changed pathways in serum between the control (CON) and label-recommended dose of antibiotic (REC) groups (**A**), and trace amounts of antibiotic (TRA) and REC groups (**B**) on d 5 post-inoculation. The x-axis represents the pathway impact values and the y-axis represents the -log(*P*) values from the pathway enrichment analysis. Metabolite set enrichment analysis (**C, D**) shows the metabolic pathways were enriched in CON compared to REC, and TRA compared to REC on d 5 post-inoculation, respectively. Both pathway analysis and metabolite set enrichment analysis were performed using identified metabolites with VIP > 1

Pathway analysis and metabolite set enrichment analysis were performed on metabolites in serum with VIP > 1. On d 0 before ETEC challenge, inositol phosphate metabolism, glyoxylate and discarboxylate metabolism, glycine, serine and threonine metabolism, citrate cycle, and ascorbate and aldarate metabolism were the most affected metabolic pathways when comparing CON with REC (Fig. S2A, C). Citrate (TCA) cycle, arginine biosynthesis, and alanine, aspartate, and glutamate metabolism were the most affected metabolic pathways when TRA was compared with REC (Fig. S2B, D). On d 5 PI, aminoacyl-tRNA biosynthesis, glycine, serine, and threonine metabolism, and phenylalanine, tyrosine, and tryptophan biosynthesis were the most affected metabolic pathways when comparing CON vs. REC (Fig. 2 A, C), while aminoacyl-tRNA biosynthesis, alanine, aspartate, and glutamate metabolism, and glycolysis and gluconeogenesis were the most affected metabolic pathways in a comparison of TRA vs. REC (Fig. 2B, D). On d 11 PI, arginine biosynthesis, alanine, aspartate and glutamate metabolism, D-glutamine and D-glutamate metabolism, pyrimidine metabolism, and citrate cycle were the most affected metabolic pathways when comparing CON with REC (Fig. S3A, C). Arginine biosynthesis, aminoacyl-tRNA biosynthesis, alanine, aspartate, and glutamate metabolism, and D-glutamine and D-glutamate metabolism were the most affected metabolic pathways in a comparison of TRA with REC (Fig. S3 B, D).

### Metabolite profiles in distal colon digesta

**Fig. 3:**
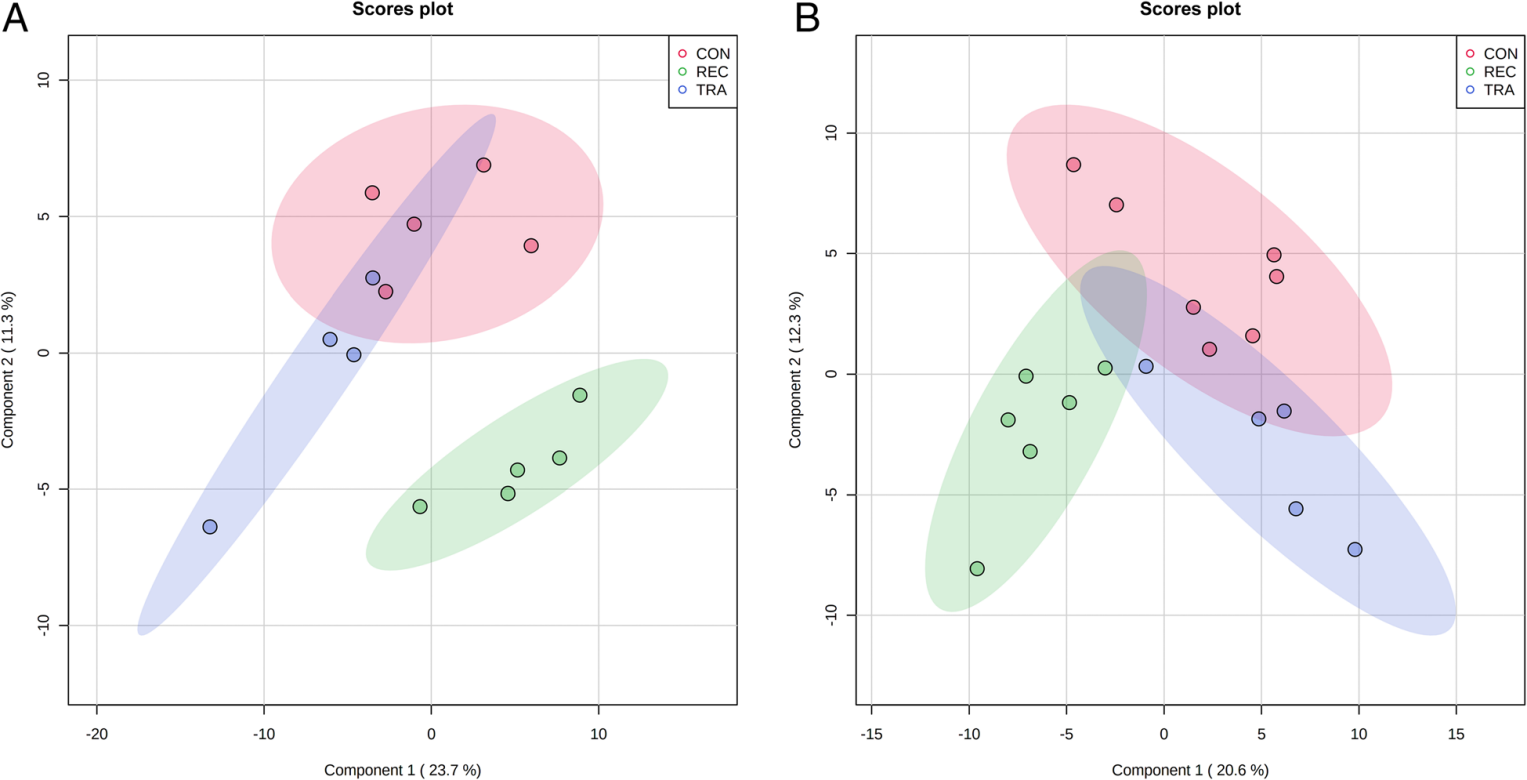
Partial Least Squares Discriminant Analysis (PLS-DA) 2D score plot of the metabolites in colon digesta showed separated clusters between the TRA and REC groups on d 5 (**A**) and 11 (**B**) post-inoculation. ● = CON (Control); ● = TRA (Trace amounts of antibiotic); ● = REC (Label-recommended dose of antibiotic). Shaded areas in different colors represent in 95% confidence interval

A total of 398 (178 identified and 220 unidentified) metabolites were detected in colon digesta. Based on VIP score and relative abundance, 12 metabolites (9 up-regulated and 3 down-regulated) were differentiated on d 5 PI, and one metabolite, inosine, was decreased on d 11 PI in pigs fed with TRA when compared with pigs in the REC group (Table 2). No differential metabolites were identified when comparing CON vs. TRA, and CON vs. REC at d 5 and 11 PI. Based on the identified metabolites, a PLS-DA score plot with 95% confidence ranges (shaded areas) showed a clear separation between the TRA and REC groups at both PI time points (Fig. 3). The PLS-DA score plots in a pairwise manner also clearly separated TRA from REC on d 5 and 11 PI (Fig. S4).

**Table 2:** Colon digesta metabolites that differed among the dietary treatment groups^1^

Metabolite	Fold change^1^	VIP^2^	FDR^3^
TRA^4^ vs. REC^5^, d 5 post-inoculation			
Octadecanol	0.38	1.82	0.173
Nonadecanoic acid	0.39	1.89	0.126
Adipic acid	0.40	1.91	0.125
Pinitol	2.22	1.82	0.173
3-hydroxy-3-methylglutaric acid	2.57	1.94	0.118
Proline	2.64	1.92	0.118
Arabitol	3.42	2.18	0.018
Lyxitol	3.92	2.16	0.018
Dehydroabietic acid	4.27	2.15	0.018
Propyleneglycol	5.09	1.92	0.118
Maltotriose	5.18	1.96	0.118
2-hydroxyvaleric acid	13.35	2.14	0.018
TRA vs. REC, d 11 post-inoculation			
Inosine	0.20	1.9793	0.160

^1^Fold change values less than one indicate that the differential metabolites were reduced in the TRA compared to REC

^2^*VIP* Variable Importance in the projection

^3^*FDR* False discovery rate

^4^*TRA* Trace amounts of antibiotic

^5^*REC* Label-recommended dose of antibiotic

**Fig. 4:**
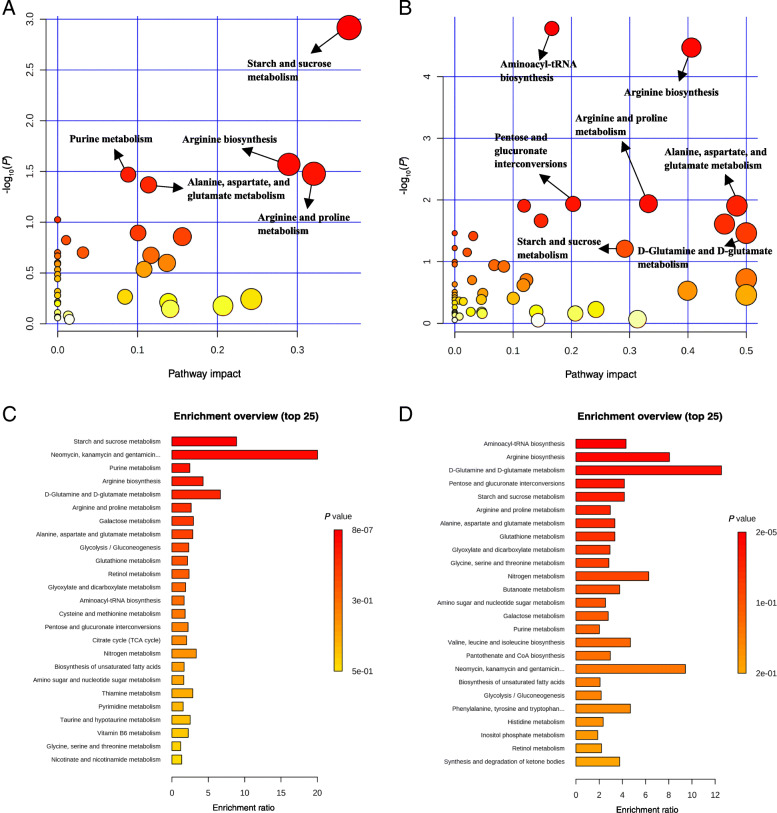
Significantly changed pathways in colon digesta between the trace amounts of antibiotic (TRA) and label-recommended dose of antibiotic (REC) groups on d 5 (**A**) and 11 post-inoculation (**B**). The x-axis represents the pathway impact values and the y-axis represents the -log(*P*) values from the pathway enrichment analysis. Metabolite set enrichment analysis (**C, D**) shows the metabolic pathways were enriched in TRA compared to REC group on d 5 and 11 post-inoculation, respectively. Both pathway analysis and metabolite set enrichment analysis were performed using identified metabolites with VIP > 1

Pathway analysis and metabolite set enrichment analysis were performed on metabolites in colon digesta with VIP > 1. Starch and sucrose metabolism, purine metabolism, arginine biosynthesis, and arginine and proline metabolism were the most affected metabolic pathways when TRA group was compared with the REC group on d 5 PI (Fig. 4 A, C). Aminoacyl-tRNA biosynthesis, arginine biosynthesis, pentose and glucuronate interconversions, arginine and proline metabolism, alanine, aspartate, and glutamate metabolism, glutathione metabolism, and glyoxylate and dicarboxylate metabolism were the most affected metabolic pathways on d 11 PI when TRA group was compared with the REC group (Fig. 4 B, D).

### Microbial profiles in distal colon digesta

A total of 481,102 qualified reads were obtained with a mean of 15,034 reads per sample. A total of 3561 OTUs were identified in the current experiment. No differences were observed in the alpha diversity of distal colon content among dietary treatments on d 5 and d 11 PI. Both Chao1 and Shannon indices of the distal colon content were lower (*P* < 0.05) on d 11 PI than d 5 PI for pigs in the CON group (Fig. S5). Beta diversity (Adonis analysis based on the Bray-Curtis distance) indicated that day (days PI) was a significant factor associated with composition distance (R^2^ = 0.11, *P* < 0.05; Fig. S6). Compositional differences of the distal colon microbiota were also observed between CON vs. REC and TRA vs. REC on d 5 and d 11 PI (Pairwise-Adonis, *P* < 0.05; Fig. S6).

**Fig. 5:**
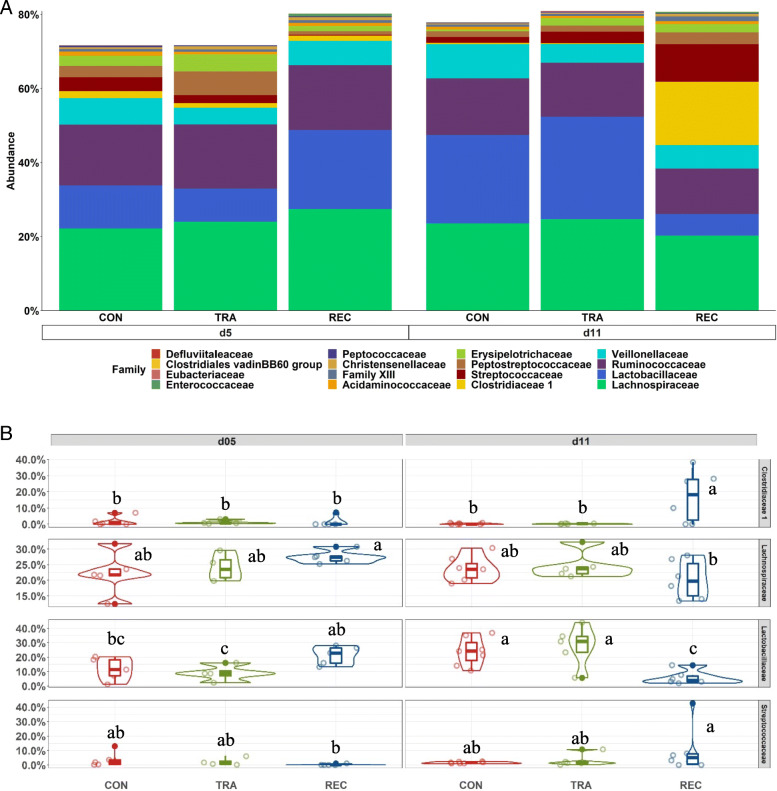
Stacked bar plot showing the relative abundance of Firmicutes family in colon digesta of enterotoxigenic *E. coli* F18 challenged pigs fed diets supplemented with different dose of antibiotic on d 5 and 11 post-inoculation (**A**). Violin plot showing the relative abundance of individual bacterial phylum (**B**). ^a-c^Means without a common superscript are different across both time points (Diet × day, *P* < 0.05). Each least squares mean represents 4 to 7 observations. CON = Control; TRA = Trace amount of antibiotic; REC = Label-recommended dose of antibiotic

**Fig. 6:**
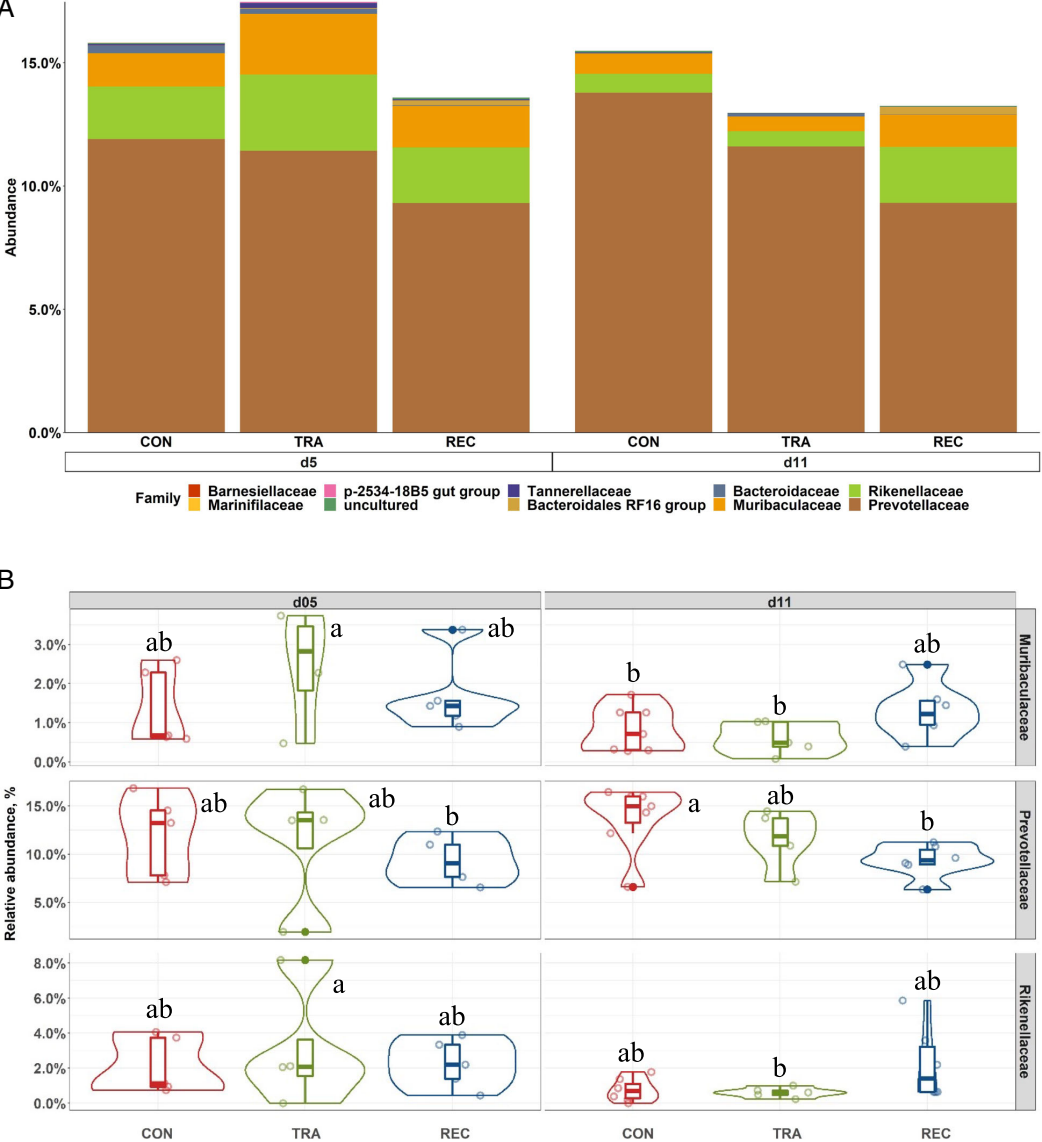
Stacked bar plot showing the relative abundance of Bacteroidetes family in colon digesta of enterotoxigenic *E. coli* F18 challenged pigs fed diets supplemented with different dose of antibiotic on d 5 and 11 post-inoculation (**A**). Violin plot showing the relative abundance of individual bacterial phylum (**B**). ^a-b^Means without a common superscript are different across both time points (Diet × day, *P* < 0.05). Each least squares mean represents 4 to 7 observations. CON = Control; TRA = Trace amount of antibiotic; REC = Label-recommended dose of antibiotic

The dominant phyla in distal colon content were Firmicutes, Bacteroidetes, Proteobacteria, and Actinobacteria, regardless of treatment or sampling day (Fig. S7). Pigs in the TRA or REC group had a lower (*P* < 0.05) relative abundance of Actinobacteria than pigs in the CON group on d 11 PI. Within the Firmicutes phylum (Fig. 5), pigs in the TRA group had lower (*P* < 0.05) relative abundance of *Lactobacillaceae* (8.91% vs. 21.33%) than pigs in REC on d 5 PI, whereas REC had lower (*P* < 0.05) relative abundance of *Lactobacillaceae* (5.82% vs. 23.90% or 27.69%) than pigs in the CON or TRA groups on d 11 PI. Pigs in the REC group had higher (*P* < 0.05) relative abundance of *Clostridiaceae* (17.14% vs. 1.45%) and *Streptococcaceae* (10.09% vs. 0.21%), but lower (*P* < 0.05) relative abundance of *Lachnospiraceae* (20.25% vs. 27.44%) in the distal colon on d 11 PI than on d 5 PI. Within the Bacteroidetes phylum (Fig. 6), pigs in the TRA group had reduced (*P* < 0.05) relative abundance of *Muribaculaceae* (0.60% vs. 2.46%) and *Rikenellaceae* (0.61% vs. 3.09%) in distal colon on d 11 PI than on d 5 PI. On d 11 PI, pigs in the CON group had higher (*P* < 0.05) relative abundance of *Prevotellaceae* (13.78% vs. 9.32%) in distal colon content, compared with pigs in the REC group.

## Discussion

In-feed antibiotics can influence nutrient metabolism and many biological processes in pigs by altering microbiota and metabolites [25, 26]. Antimicrobial agent used in present study, carbadox, is one of the most common antibiotics and widely used in the U.S. swine industry to control enteric diseases and to promote the growth of nursery pigs [27]. However, little is known about the impacts of trace amounts of antibiotics on metabolic and microbial changes in piglets, especially under disease-challenged conditions. The present study investigated the alteration of metabolic pathways in the serum and colon digesta by using an untargeted metabolomics approach when pigs were supplemented with different levels of the antibiotic carbadox. Results from the current study highlighted that supplementing label-recommended doses of antibiotics altered metabolomic markers related to nutrient metabolism in the serum and colon digesta. Moreover, supplementation of different levels of antibiotic modified microbial community composition and diversity to a different extent in the colon digesta of pigs challenged with ETEC F18. Our previous research reported that supplementing the label-recommended dose of antibiotic enhanced disease resistance and promoted growth, whereas supplementing trace amounts of antibiotic exacerbated the detrimental effects of ETEC F18 infection on performance and diarrhea, and systemic inflammation of weaned pigs [12]. Results from the current study will help us to understand the negative impacts of trace amounts of antibiotic on performance and health of young pigs by focusing on the gut microbiome and their metabolites and the host metabolism.

The metabolomics approach exploits high-throughput analytical measurements to identify host and microbiota metabolites and associated biological changes that are affected by internal or external factors to maintain homeostasis [28]. In the present study, differences in the metabolic profiles of serum and colon digesta were found predominately between pigs supplemented with trace amounts of antibiotic and label-recommended dose of antibiotic, especially during the peak infection period (d 5 PI). These findings suggest the comparative dose-response metabolic effects of antibiotics during ETEC infection in weaned pigs.

In-feed antibiotics mediate growth enhancement as a result of improved nutrient utilization in pigs. Growing evidence suggests that the administration of in-feed antibiotics can enhance nutrient digestibility and regulate the nutrient metabolism of the host [29]. The bacteriostatic activity of in-feed antibiotics may also impact the intestinal microbial metabolites by reducing growth depressing microbiota [30]. It was reported that in-feed antibiotics at a subtherapeutic concentration could enhance amino acid availability in piglets, as indicated by increased serum metabolomic markers that are associated with amino acid metabolism [25]. Amino acid metabolism is extremely important to support animal growth, maintain homeostasis, and regulate other biological processes in the host and intestinal microbiota [31, 32]. In the present study, metabolites related to amino acid metabolism (2-hydroxyvaleric acid, pipecolinic acid, histidine, and creatine) were enriched in the serum of pigs fed with the label-recommended antibiotic dose compared with pigs in the trace amounts of antibiotic group. This was likely due to the reduced peptide catabolism initiated by microbial protease activities when feeding label-recommended dose of antibiotic [33]. However, 2-hydroxyvaleric acid, a metabolomic marker of branched-chain amino acid catabolism, was observed to be reduced in the colon digesta of pigs fed with label-recommended dose antibiotic compared with the trace amounts of antibiotic group. These observations are in agreement with a previous research, in which Mu et al. [25] also reported that increased serum metabolites related to amino acid metabolism were concomitant with a decrease in jejunal metabolites associated with amino acid metabolism in pigs fed with a mixture of antibiotics at a growth-promoting dose. Thus, these results suggest that the systemic interplay between microbiota and metabolite profiles was promoted by feeding label-recommended dose of antibiotics. A previous study using metagenomic analysis also observed that antibiotics at subtherapeutic doses reduced the abundance of clusters of orthologous groups involved in protein metabolism in the fecal microbiota of pigs [34]. Consistent with performance data and clinical signs [12], it is not surprising to observe that trace amount of antibiotics had different impacts on serum and colon digesta metabolites that are associated with amino acid metabolism when compared with label-recommended dose of antibiotic. Previous in vitro research suggested that *E. coli* cells stimulated cellular functions and metabolic modifications of amino acid catabolism upon exposure to the antibiotic ampicillin at below the minimal inhibitory concentrations (sub-MIC) [35]. More specifically, *E. coli* cells treated with sub-MIC ampicillin resulted in increased amino acid depletion in Luria-Bertani (LB) media due to stress responses, which provided amino acids as a major energy source for cultured cells. This finding indicates that the alteration of metabolomic markers of amino acid metabolism caused by trace amounts of antibiotic in the current study may be related to the depletion of amino acids during the host response to ETEC infection. Subsequently, less amounts of amino acids might be available to support the growth of the pigs when they were challenged with ETEC and supplemented with trace amount of antibiotics.

Carbohydrate metabolism is essential to support the virulence of pathogenic enterobacteria [36]. It has been reported that the colonization of pathogenic *E. coli* in the mouse intestine was supported by the catabolism of several carbohydrates, including galactose, fucose, mannose, and maltose [37, 38]. In the present study, metabolomic markers related to galactose metabolism (glycerol and myo-inositol) and carbohydrate digestion and absorption (maltotriose) were enriched in the serum or colon digesta from pigs supplemented with trace amounts of antibiotic. This finding suggests that trace amounts of antibiotic may assist in constitution of an ecological niche for ETEC F18 colonization in the intestine of pigs, rather than exhibit its antibacterial activity. Besides the carbohydrate utilization by pathogens to colonize, carbohydrate metabolism is also vital for the systemic inflammatory response [39]. Baurhoo et al. [40] reported that a significant mobilization and catabolism of carbohydrates were observed in the intestine of chickens during LPS-induced systemic inflammation. In the present animal study, trace amounts of antibiotic exacerbated the intestinal and systemic inflammatory status of ETEC F18 challenged pigs [12]. Thus, the increased metabolites associated with carbohydrate metabolism in pigs supplemented with trace amount of antibiotics during the peak of ETEC infection indicates that these pigs may utilize more carbohydrates as energy sources to support their immune responses and recovery processes against ETEC F18 instead of growth.

Interestingly, supplementation of trace amounts of antibiotic also enriched serum metabolomic markers of purine metabolism (hypoxanthine and guanine) during the peak of ETEC infection. A previous in vitro study reported that *Pasteurella multocida* significantly increased the expression of proteins involved in purine synthesis and metabolism, in response to sub-MIC antibiotics, including amoxicillin, chlortetracycline, and enrofloxacin [41]. Ng et al. [42] also demonstrated that extremely low concentrations of antibiotics, such as tetracycline and macrolide, upregulated the expression of genes associated with purine metabolism in *Streptococcus pneumoniae*. The metabolites involved in purine metabolism are often upregulated in the activated immune cells as important immune signaling molecules [43]. For instance, previous research reported that mice infected with *E. coli* had enriched plasma metabolites that are linked to the purine metabolic pathway [44]. Likewise, growing evidence also suggests that trace concentrations of antibiotics may perform as signaling agents and trigger special bacterial responses, such as increased purine metabolism, following an infection [43, 45], Thus, our results indicate that purine metabolism might contribute to the elevated systemic inflammation in pigs fed with trace amounts of antibiotic [12].

The composition and diversity of gut microbial communities in pigs are greatly impacted by their age, health status, and nutrient components in feed [46–48]. To test the impacts of trace amounts of antibiotic on gut microbiota diversity, distal colon contents were collected, and 16S rRNA gene sequencing was performed. Consistent with previously published research, antibiotics-treated at recommended concentrations clustered separately from non-treated groups [34, 49], indicating that antibiotics administration at label-recommended dose altered colon microbiota composition and diversity. However, there was no clear separation in distal colon microbiota between pigs supplemented with trace amounts of antibiotic and pigs in the control group.

The three most abundant phyla found in the colon digesta of pigs in the present study were Firmicutes, Bacteroidetes, and Proteobacteria, which was consistent with previously published research [50, 51]. Within the Firmicutes and Bacteroidetes phyla, the relative abundance of *Lachnospiraceae* and *Lactobacillaceae* were enriched in the distal colon of pigs supplemented with label-recommended dose of antibiotic, but the relative abundance of *Lactobacillaceae* was reduced in colon digesta of trace amounts of antibiotic pigs during the peak infection period. *Lachnospiraceae* family contain numerous genera involved in producing butyric acid, which provides energy for other microbes and host epithelial cells and prevents the growth of other microbes in the digestive tract [52, 53]. Moreover, *Lactobacillaceae* were reported to be positively correlated with feed efficiency [54] and nitrogen, energy, cellulose, and hemicellulose digestibility in pigs [55]. Although the exact mechanism of antimicrobial effects is not yet clear, *Lactobacillaceae* are known for their health-promoting effects and for their ability to inhibit intestinal pathogens such as *E. coli* and *Salmonella* [56]. Thus, *Lachnospiraceae* and *Lactobacillaceae* have been proposed and investigated as biomarkers to predict the health status of pigs [57, 58]. Rhouma et al. [59] demonstrated that the ETEC F4 challenge suppressed the relative abundance of *Lachnospiraceae* and *Lactobacillaceae* in fecal contents of pigs, compared with unchallenged pigs. In addition, Dou et al. [60] also reported that diarrheic pigs, in natural post-weaning diarrhea, had a lower abundance of *Lachnospiraceae* and *Lactobacillaceae* in feces, compared with healthy pigs. Therefore, the modified intestinal microbial environment, including the enhanced presence of *Lachnospiraceae* and *Lactobacillaceae*, may contribute to the accelerated recovery from ETEC 18 infection in pigs supplemented with label-recommended dose of antibiotics.

Previous studies have also reported the contribution of intestinal microbiota to weight gain in pigs. For example, Kim et al. [61] observed that *Clostridiaceae* in the distal gut of pigs were positively correlated with weight gain, while Unno et al. [62] reported a negative correlation between weight and *Prevotellaceae* in feces when pigs were supplemented with different types of antibiotics. In the present study, pigs fed label-recommended dose antibiotic had increased relative abundance of *Clostridiaceae* but reduced relative abundance of *Prevotellaceae* compared to pigs in the control group on d 11 PI. These observations are consistent with the literature and confirmed the effectiveness of label-recommended dose of antibiotic for growth-promoting purposes [12].

In conclusion, the metabolomics and microbiome approaches in the present study identified the differential metabolites and their pathways in the serum and distal colon digesta of ETEC F18 challenged pigs. Compared with label-recommended dose of antibiotic, trace amounts of antibiotic oppositely affected metabolomic markers related to the metabolisms of amino acids, carbohydrates, and purine. Pigs administered label-recommended dose of antibiotic had marked modulation of microbial composition, which may be highly correlated with their enhanced growth performance and disease resistance in weaned pigs. The impacted metabolic pathways and colonic microbial shift may also be closely associated with the slow growth and delayed recovery from ETEC infection of weaned pigs supplemented with trace amounts of antibiotic. Future studies will consider incorporating targeted metabolomics and metagenomics to provide more insights into the potential risk of trace amounts of antibiotic on the host response to ETEC infection. The exploration of metabolomic markers and gut microbiome interaction will be important to decipher the mechanisms of how trace amounts of antibiotic negatively impact the health of young animals.

## Supplementary Information

**Additional file 1: Fig. S1.** Partial Least Squares Discriminant Analysis (PLS-DA) 2D score plot of the metabolites in serum revealed significant differences on d 0 before *E. coli* challenge, d 5 and 11 post-inoculation between the TRA and REC groups (**A-C**) and CON and REC (**D-E**), respectively. ● = CON (Control); ● = TRA (Trace amounts of antibiotic); ● = REC (Label-recommended dose of antibiotic). Shaded areas in different colors represent in 95% confidence interval. **Fig. S2.** Significantly changed pathways in serum between the control (CON) and label-recommended dose of antibiotic (REC) groups (**A**), and trace amounts of antibiotic (TRA) and REC groups (**B**) on d 0 before *E. coli* challenge. The x-axis represents the pathway impact values and the y-axis represents the -log(P) values from the pathway enrichment analysis. Metabolite set enrichment analysis (**C, D**) shows the metabolic pathways were enriched in CON compared to REC, and TRA compared to REC on d 0 before *E. coli* challenge, respectively. Both pathway analysis and metabolite set enrichment analysis were performed using identified metabolites with VIP > 1. **Fig. S3.** Significantly changed pathways in serum between the control (CON) and label-recommended dose of antibiotic (REC) groups (**A**), and trace amounts of antibiotic (TRA) and REC groups (**B**) on d 11 post-inoculation. The x-axis represents the pathway impact values and the y-axis represents the -log(P) values from the pathway enrichment analysis. Metabolite set enrichment analysis (**C, D**) shows the metabolic pathways were enriched in CON compared to REC, and TRA compared to REC on d 11 post-inoculation, respectively. Both pathway analysis and metabolite set enrichment analysis were performed using identified metabolites with VIP > 1. **Fig. S4.** Partial Least Squares Discriminant Analysis (PLS-DA) 2D score plot of the metabolites in colon digesta revealed significant differences between the TRA and REC groups on d 5 (**A**) and 11 (**B**) post-inoculation. ● = TRA (Trace amounts of antibiotic); ● = REC (Label-recommended dose of antibiotic). Shaded areas in different colors represent in 95% confidence interval. **Fig. S5.** Alpha diversity as indicated by Chao 1 (**A**) and Shannon (**B**) indices in colon digesta of enterotoxigenic *E. coli* F18 challenged pigs fed diets supplemented with different dose of antibiotic on d 5 and 11 post-inoculation. ^a-b^Means without a common superscript are different across both time points (Diet × day, *P* < 0.05). Each least squares mean represents 4 to 7 observations. CON = Control; TRA = Trace amount of antibiotic; REC = Label-recommended dose of antibiotic. **Fig. S6.** Beta diversity of colon digesta of enterotoxigenic *E. coli* F18 challenged pigs fed diets supplemented with different dose of antibiotic on d 5 and 11 post-inoculation. Data were analyzed by principal coordinate analysis (PCoA) based on the Bray-Curtis dissimilarity. Symbols indicate dietary treatments and colors indicate different sampling dates. CON = Control; TRA = Trace amount of antibiotic; REC = Label-recommended dose of antibiotic. **Fig. S7.** Stacked bar plot showing the relative abundance of bacterial phyla in colon digesta of enterotoxigenic *E. coli* F18 challenged pigs fed diets supplemented with different dose of antibiotic on d 5 and 11 post-inoculation (**A**). Violin plot showing the relative abundance of individual bacterial phylum (**B**). ^a-c^Means without a common superscript are different across both time points (Diet × day, *P* < 0.05). Each least squares mean represents 4 to 7 observations. CON = Control; TRA = Trace amount of antibiotic; REC = Label-recommended dose of antibiotic.

## Data Availability

All data generated or analyzed during this study are available from the corresponding author upon reasonable request.

## Abbreviations

FDR: False discovery rate
ETEC: Enterotoxigenic *E. coli*
MIC: Minimal inhibitory concentrations
OTU: Operational taxonomic units
PI: Post-inoculation
PLS-DA: Partial least squares discriminant analysis
PCoA: Principal coordinates analysis
VIP: Variable importance in the projection
