# Supplementary material for: Trace amounts of antibiotic altered metabolomic and microbial profiles of weaned pigs infected with a pathogenic E. coli
Source: J Anim Sci Biotechnol. 2022 May 9;13:59. doi: 10.1186/s40104-022-00703-5 (PMC9082874; doi:10.1186/s40104-022-00703-5)
Supplement: Supplementary file 1 — Additional file 1: Fig. S1. Partial Least Squares Discriminant Analysis (PLS-DA) 2D score plot of the metabolites in serum revealed significant differences on d 0 before E. coli challenge, d 5 and 11 post-inoculation between the TRA and REC groups (A-C) and CON and REC (D-E), respectively. ● = CON (Control); ● = TRA (Trace amounts of antibiotic); ● = REC (Label-recommended dose of antibiotic). Shaded areas in different colors represent in 95% confidence interval. Fig. S2. Significantly changed pathways in serum between the control (CON) and label-recommended dose of antibiotic (REC) groups (A), and trace amounts of antibiotic (TRA) and REC groups (B) on d 0 before E. coli challenge. The x-axis represents the pathway impact values and the y-axis represents the -log(P) values from the pathway enrichment analysis. Metabolite set enrichment analysis (C, D) shows the metabolic pathways were enriched in CON compared to REC, and TRA compared to REC on d 0 before E. coli challenge, respectively. Both pathway analysis and metabolite set enrichment analysis were performed using identified metabolites with VIP > 1. Fig. S3. Significantly changed pathways in serum between the control (CON) and label-recommended dose of antibiotic (REC) groups (A), and trace amounts of antibiotic (TRA) and REC groups (B) on d 11 post-inoculation. The x-axis represents the pathway impact values and the y-axis represents the -log(P) values from the pathway enrichment analysis. Metabolite set enrichment analysis (C, D) shows the metabolic pathways were enriched in CON compared to REC, and TRA compared to REC on d 11 post-inoculation, respectively. Both pathway analysis and metabolite set enrichment analysis were performed using identified metabolites with VIP > 1. Fig. S4. Partial Least Squares Discriminant Analysis (PLS-DA) 2D score plot of the metabolites in colon digesta revealed significant differences between the TRA and REC groups on d 5 (A) and 11 (B) post-inoculation. ● = TRA (Trace amounts [file 40104_2022_703_MOESM1_ESM.docx]

**Figure S1.** Partial Least Squares Discriminant Analysis (PLS-DA) 2D score plot of the metabolites in serum revealed significant differences on d 0 before *E. coli* challenge, d 5 and 11 post-inoculation between the TRA and REC groups (A-C) and CON and REC (D-E), respectively. ● = CON (Control); ● = TRA (Trace amounts of antibiotic); ● = REC (Label-recommended dose of antibiotic). Shaded areas in different colors represent in 95% confidence interval.


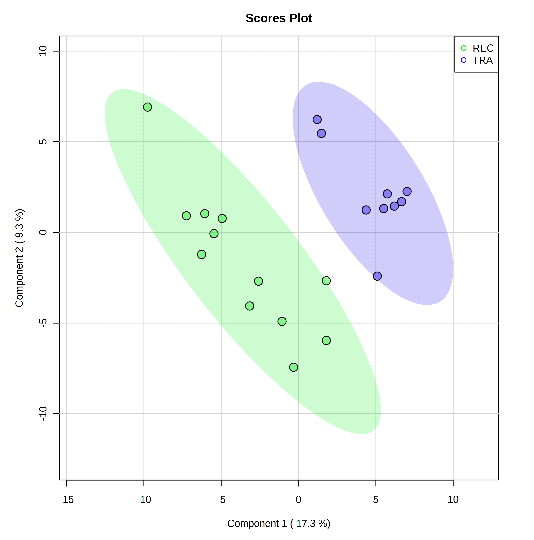

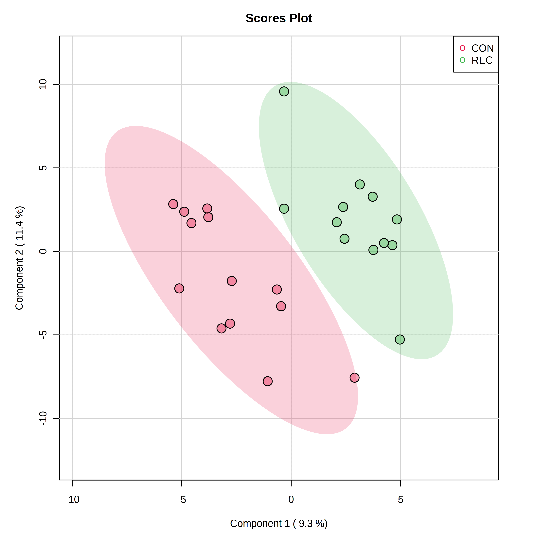

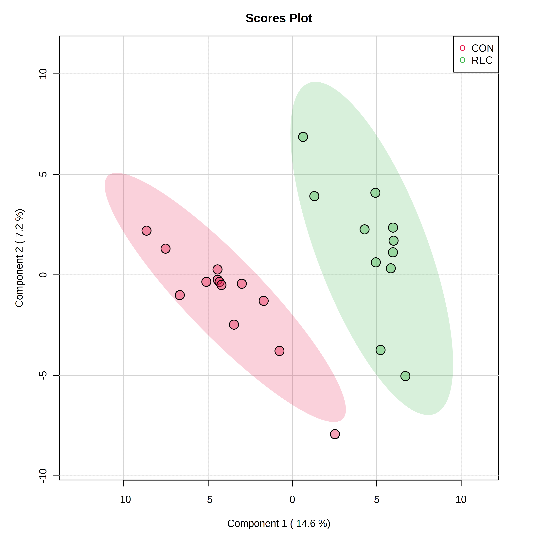

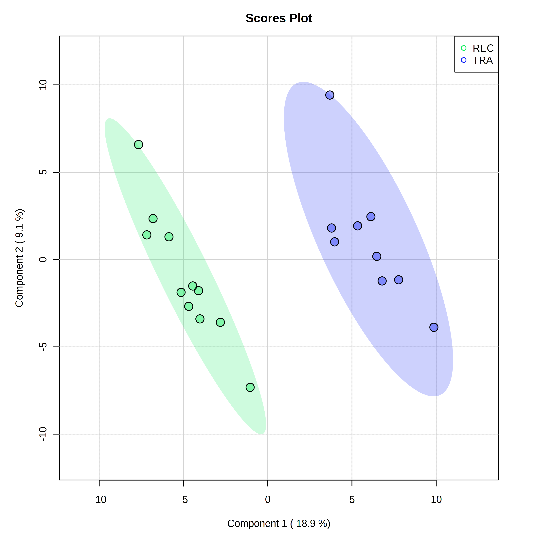

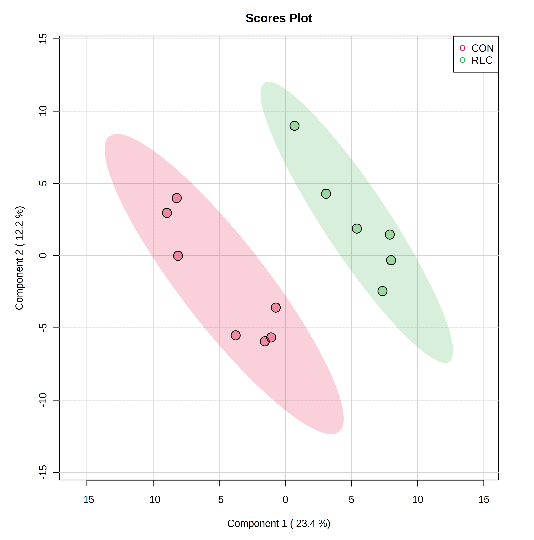

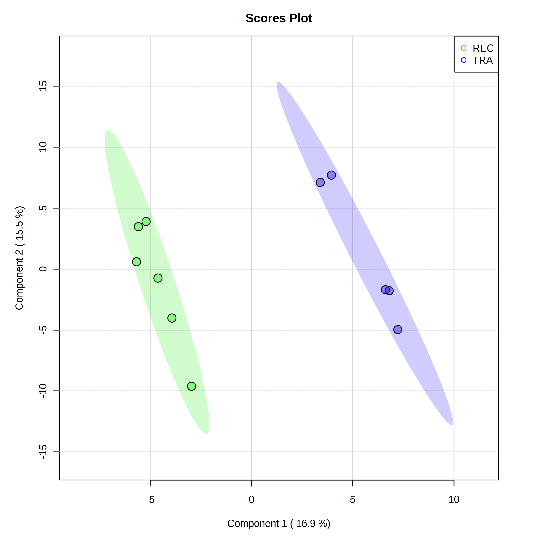


**A**

**B**

**C**

**D**

**E**

**F**

**Figure S2.** Significantly changed pathways in serum between the control (CON) and label-recommended dose of antibiotic (REC) groups (A), and trace amounts of antibiotic (TRA) and REC groups (B) on d 0 before *E. coli* challenge. The x-axis represents the pathway impact values and the y-axis represents the -log(P) values from the pathway enrichment analysis. Metabolite set enrichment analysis (C, D) shows the metabolic pathways were enriched in CON compared to REC, and TRA compared to REC on d 0 before *E. coli* challenge, respectively. Both pathway analysis and metabolite set enrichment analysis were performed using identified metabolites with VIP > 1.


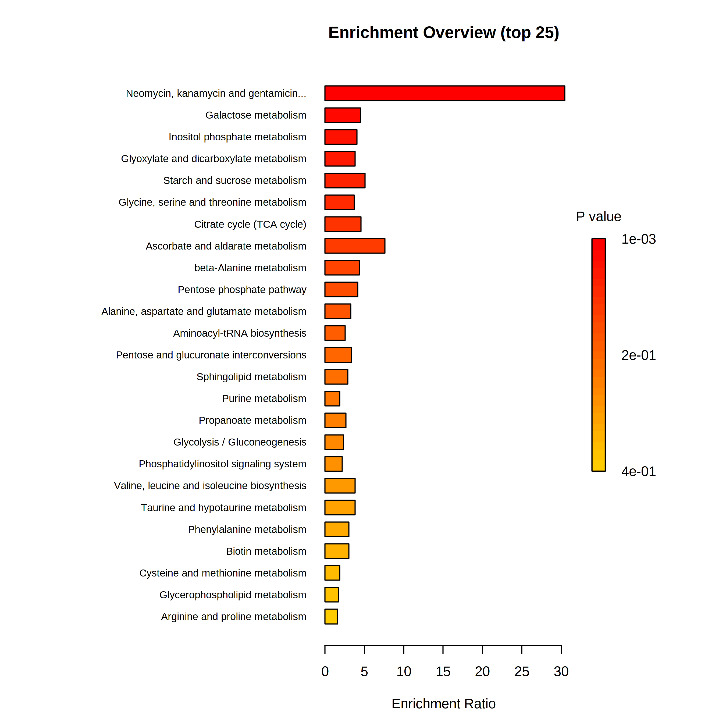

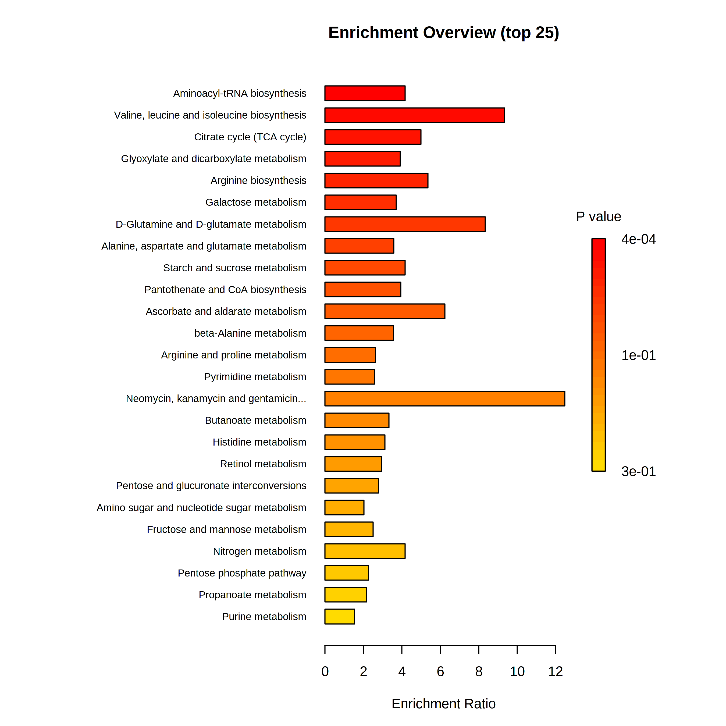

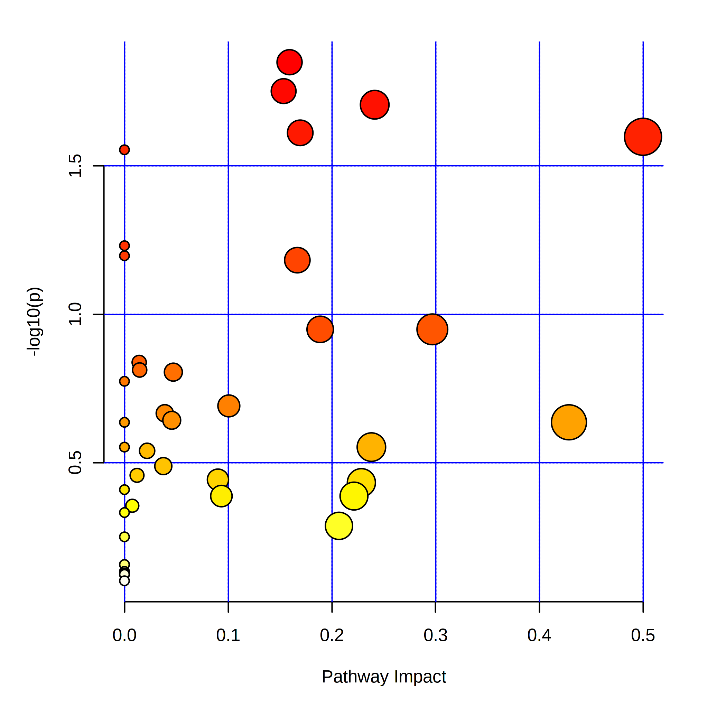


**A**

**Inositol phosphate metabolism**

**Glyoxylate and discarboxylate metabolism**

**Citrate cycle (TCA cycle)**

**Glycine, serin and threonine metabolism**

**Ascorbate and aldarate metabolism**


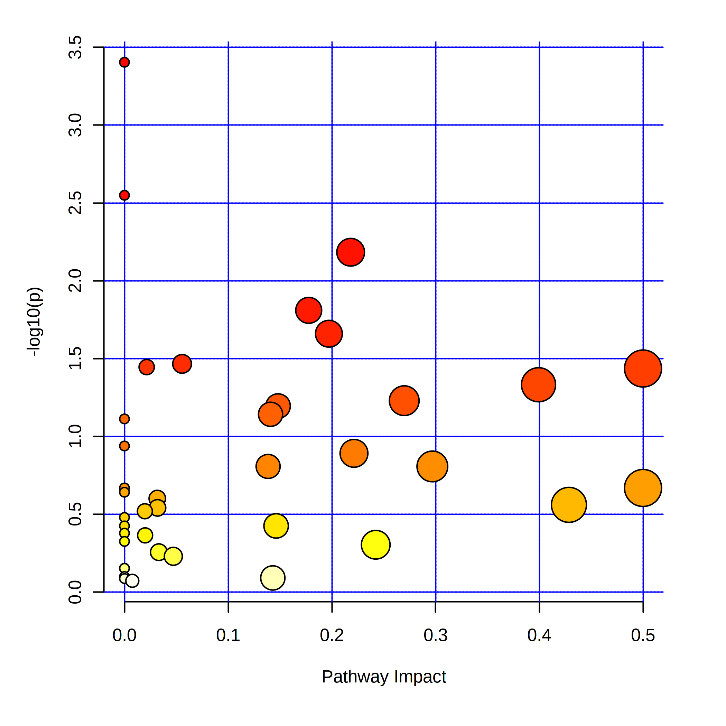


**B**

**Citrate cycle (TCA cycle)**

**Arginine biosynthesis**

**Alanine, aspartate, and glutamate metabolism**

**C**

**D**

**Figure S3.** Significantly changed pathways in serum between the control (CON) and label-recommended dose of antibiotic (REC) groups (A), and trace amounts of antibiotic (TRA) and REC groups (B) on d 11 post-inoculation. The x-axis represents the pathway impact values and the y-axis represents the -log(P) values from the pathway enrichment analysis. Metabolite set enrichment analysis (C, D) shows the metabolic pathways were enriched in CON compared to REC, and TRA compared to REC on d 11 post-inoculation, respectively. Both pathway analysis and metabolite set enrichment analysis were performed using identified metabolites with VIP > 1.


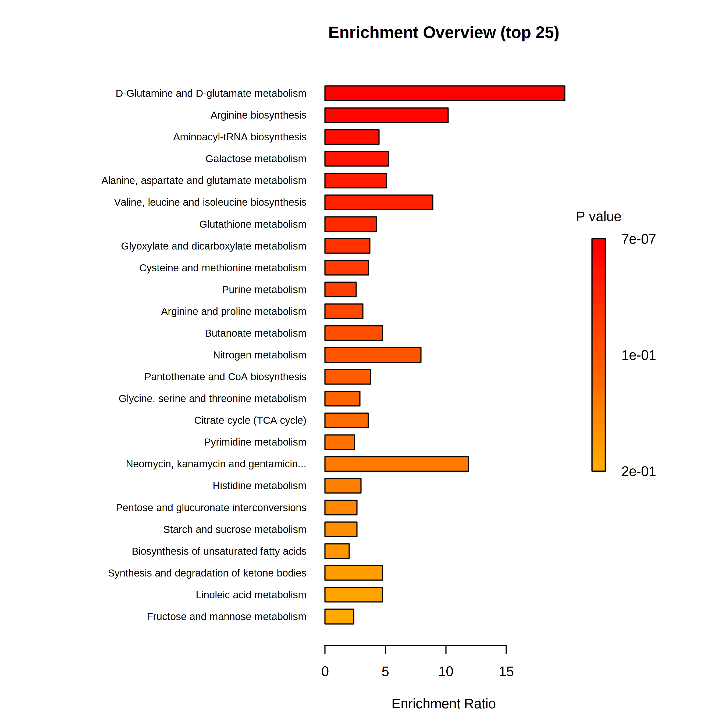

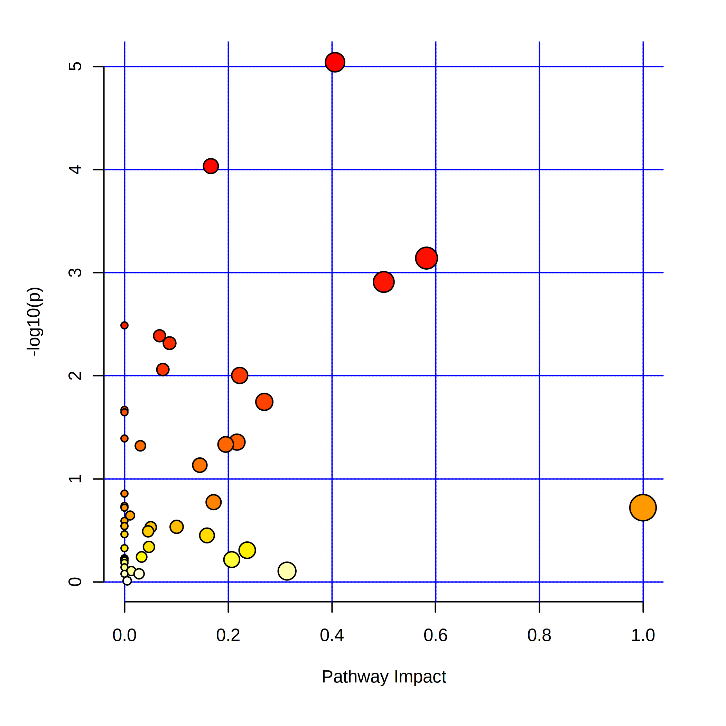

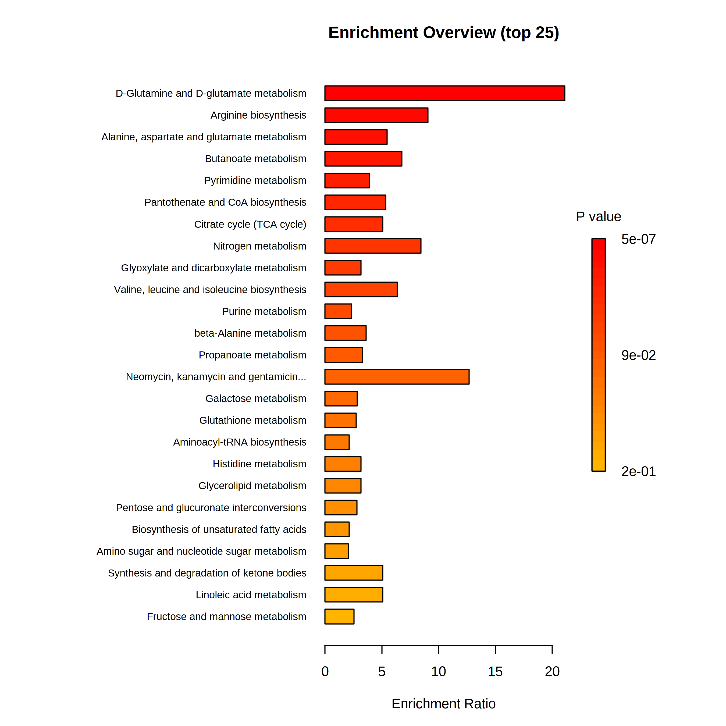

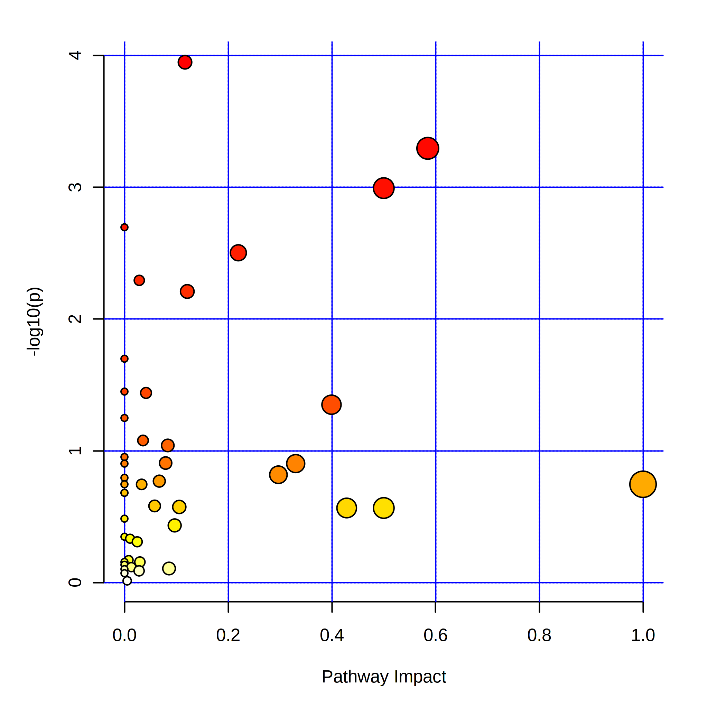


**B**

**C**

**D**

**A**

**Arginine biosynthesis**

**Alanine, aspartate, and glutamate metabolism**

**D-Glutamine and D-glutamate metabolism**

**Pyrimidine metabolism**

**Citrate cycle (TCA cycle)**

**Arginine biosynthesis**

**Aminoacyl-tRNA biosynthesis**

**Alanine, aspartate, and glutamate metabolism**

**D-Glutamine and D-glutamate metabolism**

**A B**


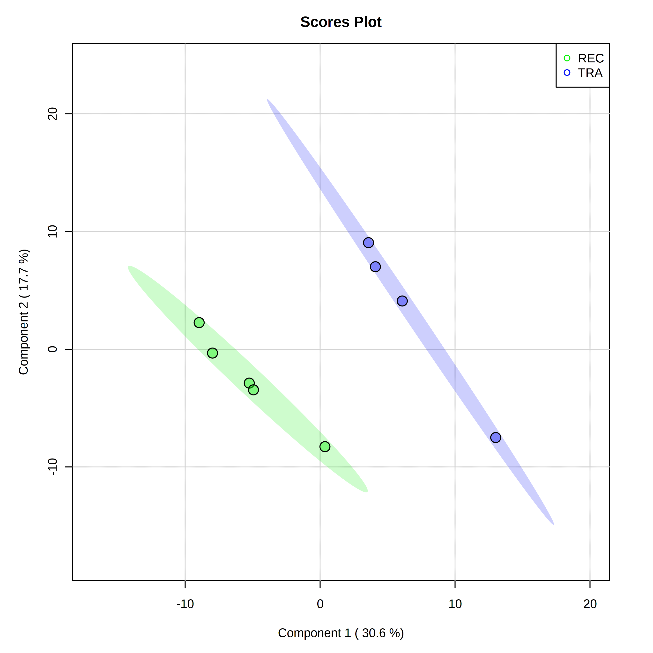

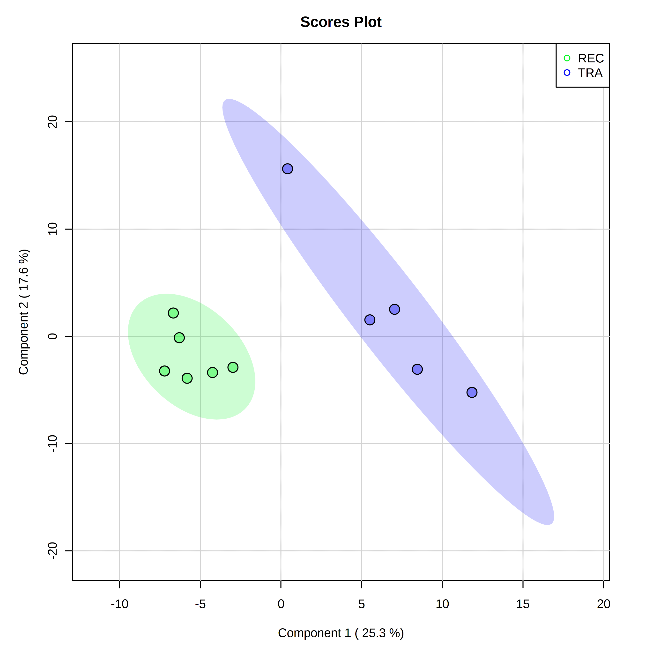


**Figure S4.** Partial Least Squares Discriminant Analysis (PLS-DA) 2D score plot of the metabolites in colon digesta revealed significant differences between the TRA and REC groups on d 5 (A) and 11 (B) post-inoculation. ● = TRA (Trace amounts of antibiotic); ● = REC (Label-recommended dose of antibiotic). Shaded areas in different colors represent in 95% confidence interval.


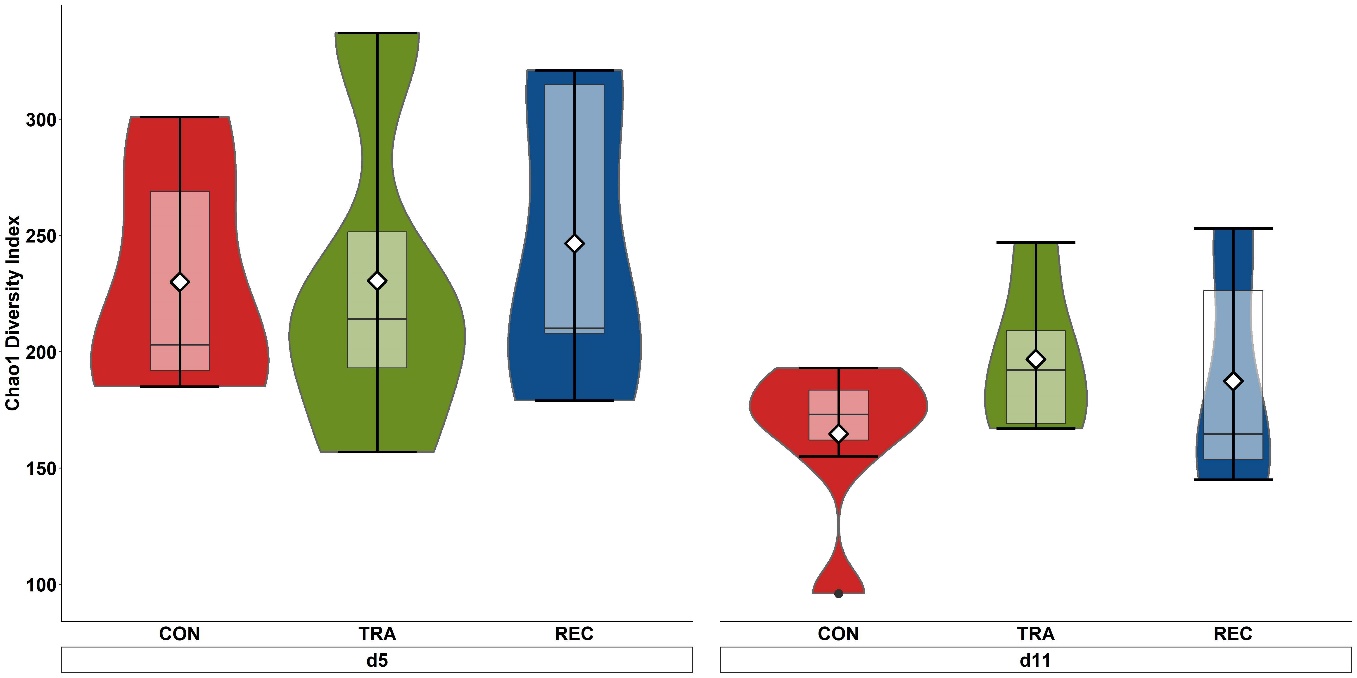


a

ab

a

b

ab

ab


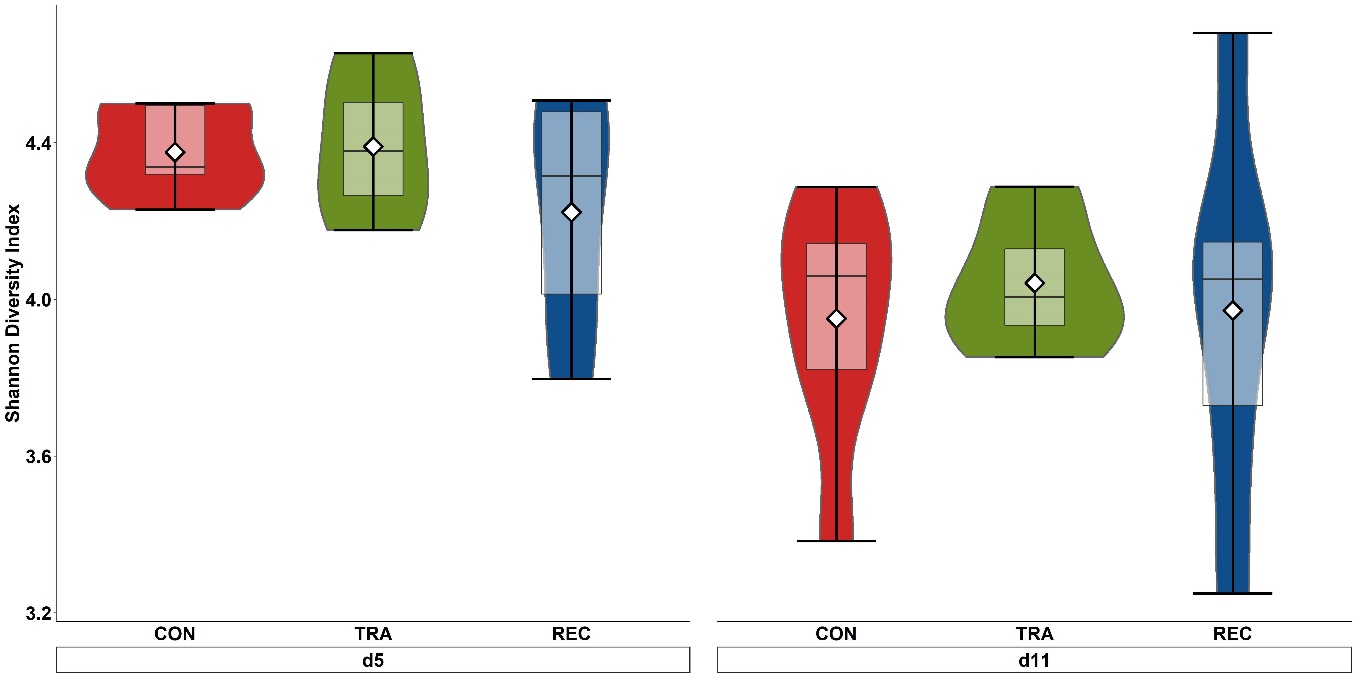


a

a

ab

b

ab

b

**A**

**B**

**Figure S5.** Alpha diversity as indicated by Chao 1 (A) and Shannon (B) indices in colon digesta of enterotoxigenic E. coli F18 challenged pigs fed diets supplemented with different dose of antibiotic on d 5 and 11 post-inoculation. ^a-b^Means without a common superscript are different across both time points (Diet × day, P < 0.05). Each least squares mean represents 4 to 7 observations. CON = Control; TRA = Trace amount of antibiotic; REC = Label-recommended dose of antibiotic.

**Figure S6.*
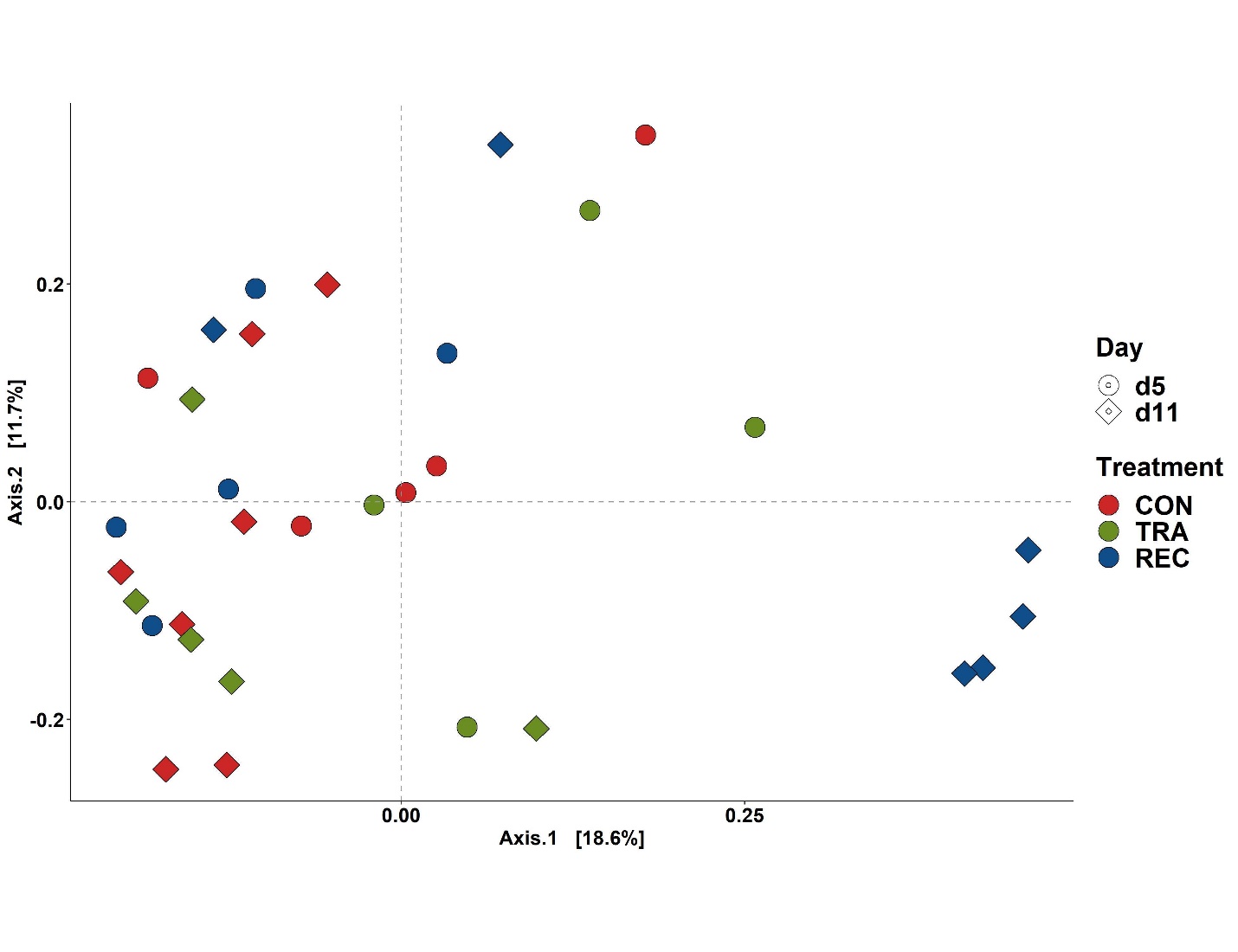
*** Beta diversity of colon digesta of enterotoxigenic E. coli F18 challenged pigs fed diets supplemented with different dose of antibiotic on d 5 and 11 post-inoculation. Data were analyzed by principal coordinate analysis (PCoA) based on the Bray-Curtis dissimilarity. Symbols indicate dietary treatments and colors indicate different sampling dates. CON = Control; TRA = Trace amount of antibiotic; REC = Label-recommended dose of antibiotic.


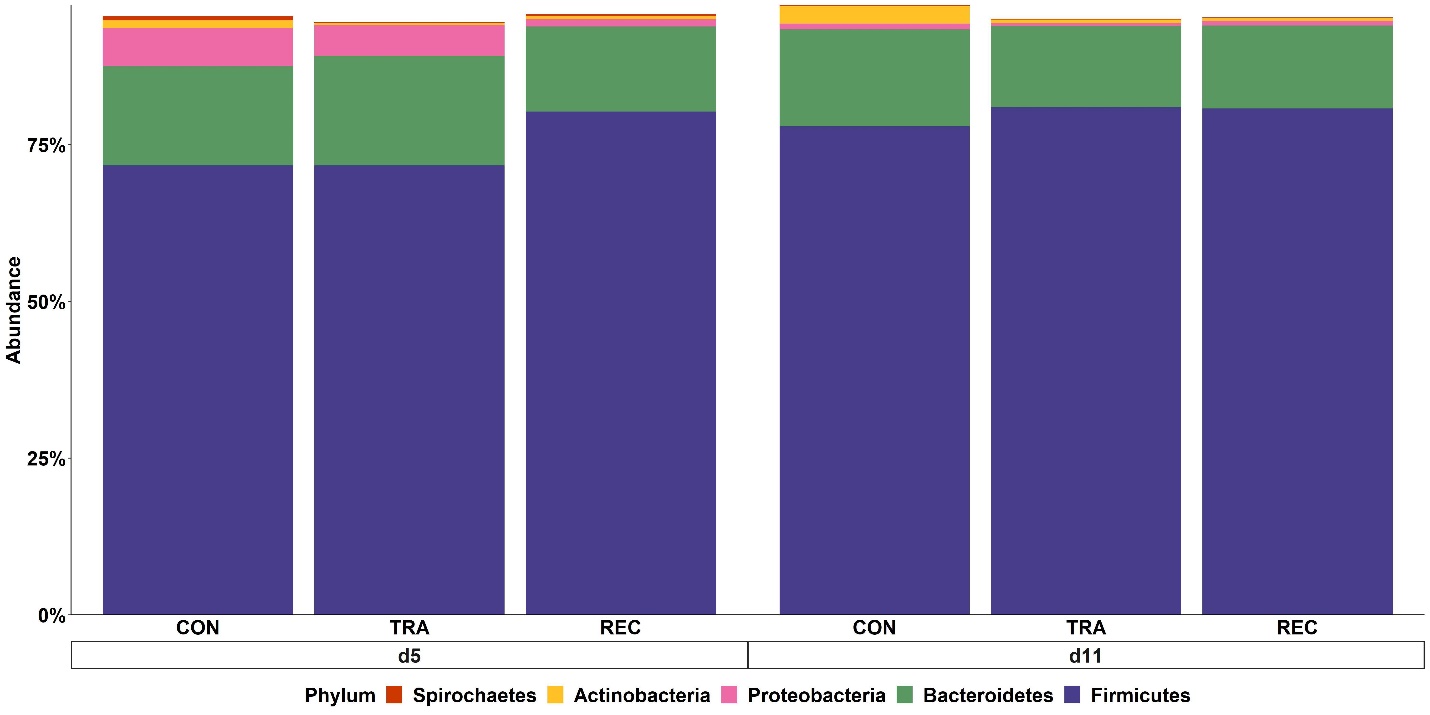

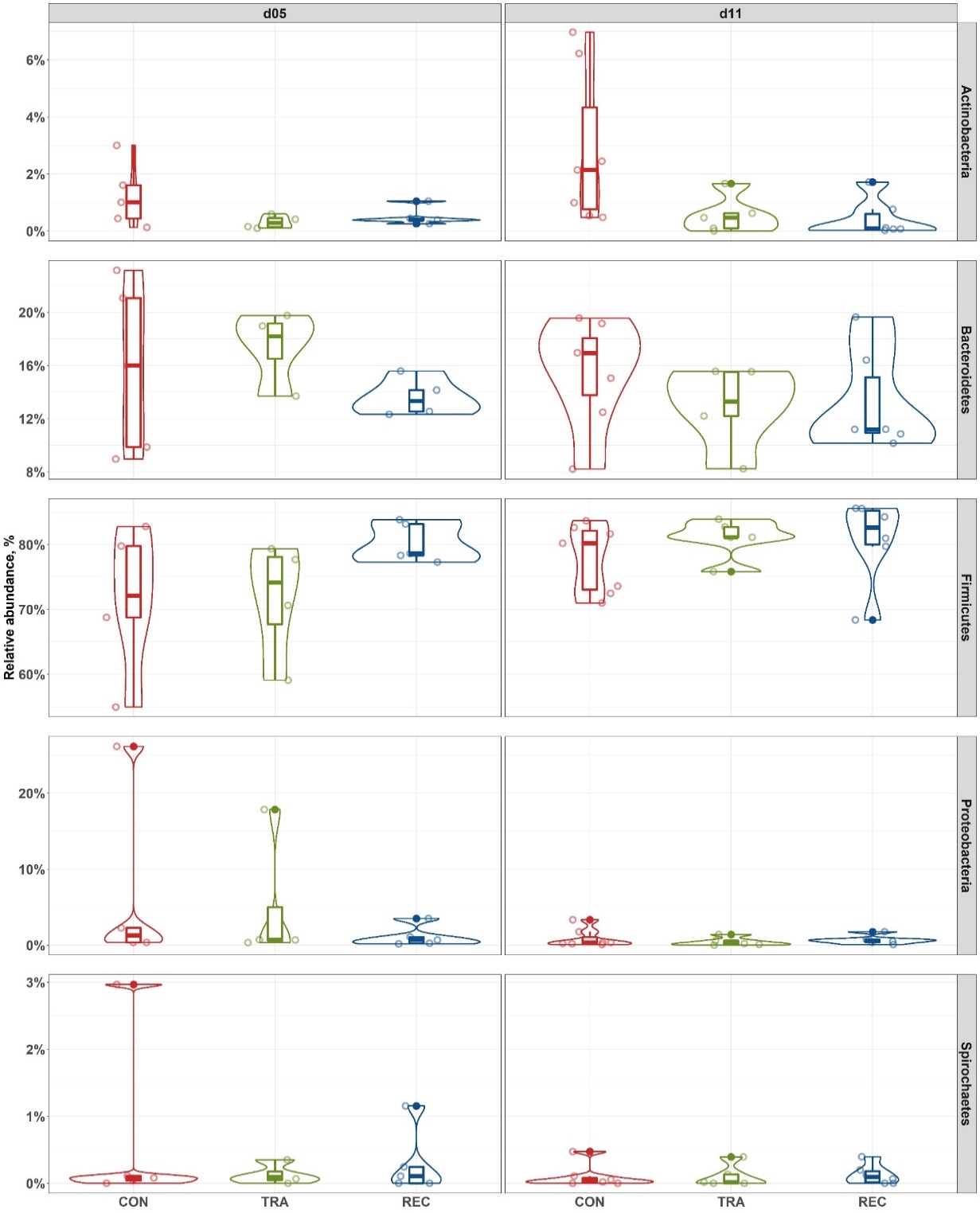

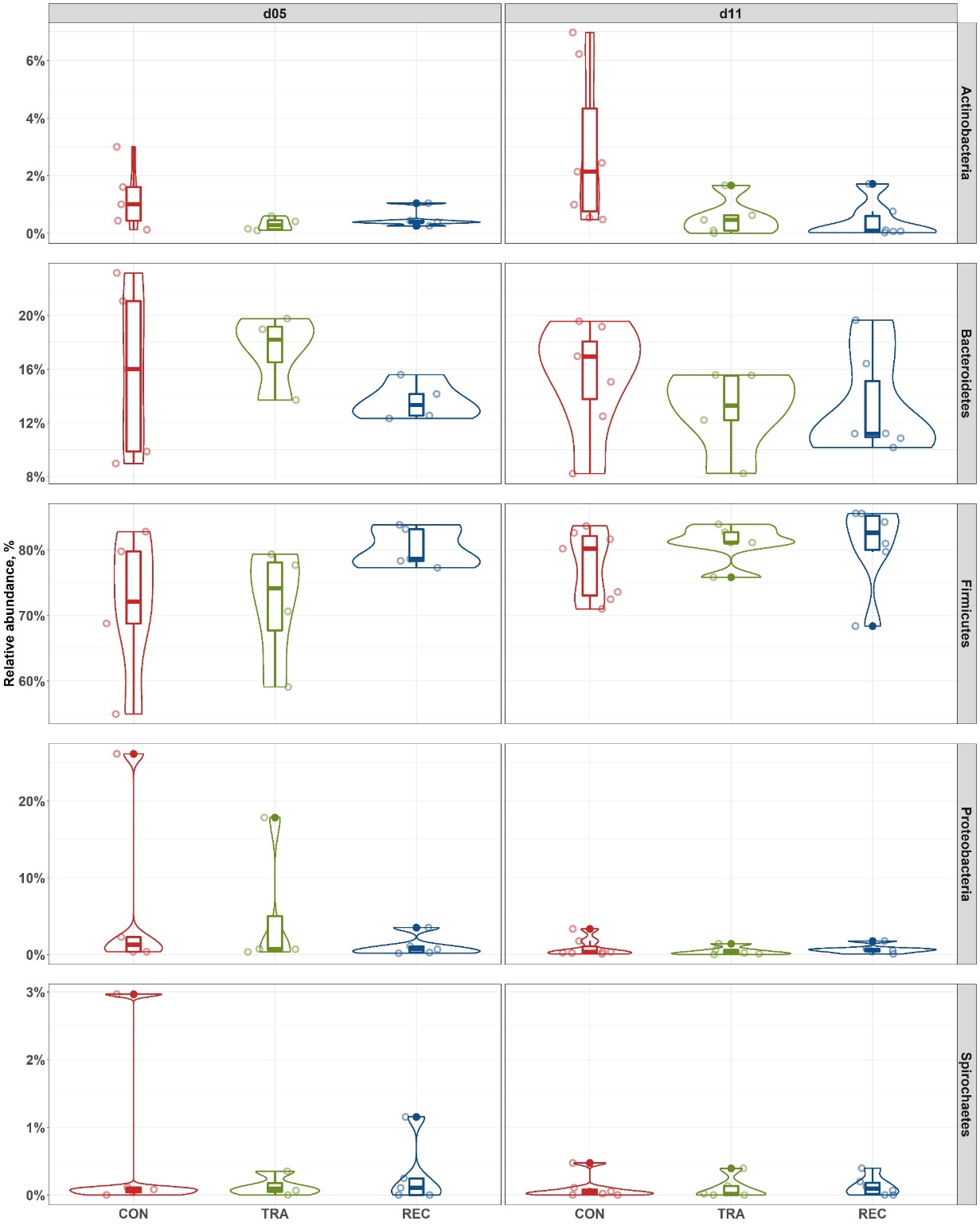


ab

b

b

a

b

b

c

bc

abc

abc

ab

a

**A**

**B**

**Figure S7.** Stacked bar plot showing the relative abundance of bacterial phyla in colon digesta of enterotoxigenic E. coli F18 challenged pigs fed diets supplemented with different dose of antibiotic on d 5 and 11 post-inoculation (A). Violin plot showing the relative abundance of individual bacterial phylum (B). ^a-c^Means without a common superscript are different across both time points (Diet × day, P < 0.05). Each least squares mean represents 4 to 7 observations. CON = Control; TRA = Trace amount of antibiotic; REC = Label-recommended dose of antibiotic.
